# Hybrid Breakdown in Cichlid Fish

**DOI:** 10.1371/journal.pone.0127207

**Published:** 2015-05-21

**Authors:** Rike Bahati Stelkens, Corinne Schmid, Ole Seehausen

**Affiliations:** 1 Max Planck Institute for Evolutionary Biology, Plön, Germany; 2 Department of Aquatic Ecology and Macroevolution, Institute of Ecology and Evolution, University of Bern, Bern, Switzerland; 3 Department of Fish Ecology and Evolution, Centre of Ecology, Evolution and Biogeochemistry (CEEB), Swiss Federal Institute of Aquatic Science and Technology (EAWAG), Kastanienbaum, Switzerland; School of Environment & Life Sciences, University of Salford, UNITED KINGDOM

## Abstract

Studies from a wide diversity of taxa have shown a negative relationship between genetic compatibility and the divergence time of hybridizing genomes. Theory predicts the main breakdown of fitness to happen after the F1 hybrid generation, when heterosis subsides and recessive allelic (Dobzhansky-Muller) incompatibilities are increasingly unmasked. We measured the fitness of F2 hybrids of African haplochromine cichlid fish bred from species pairs spanning several thousand to several million years divergence time. F2 hybrids consistently showed the lowest viability compared to F1 hybrids and non-hybrid crosses (crosses within the grandparental species), in agreement with hybrid breakdown. Especially the short- and long-term survival (2 weeks to 6 months) of F2 hybrids was significantly reduced. Overall, F2 hybrids showed a fitness reduction of 21% compared to F1 hybrids, and a reduction of 43% compared to the grandparental, non-hybrid crosses. We further observed a decrease of F2 hybrid viability with the genetic distance between grandparental lineages, suggesting an important role for negative epistatic interactions in cichlid fish postzygotic isolation. The estimated time window for successful production of F2 hybrids resulting from our data is consistent with the estimated divergence time between the multiple ancestral lineages that presumably hybridized in three major adaptive radiations of African cichlids.

## Introduction

The loss of genetic compatibility is one of the causes of reproductive isolation in classical models of speciation [1]. Hybridization between divergent populations or species is thought to result in hybrid offspring with decreased fitness, and different genetic models for the accumulation of such incompatibilities have been proposed. The Dobzhansky-Muller (D-M) model is the most widely accepted. In the D-M model, alleles with neutral or beneficial effects in one population reduce offspring fitness when recombined in a hybrid genome with alleles originating at different loci in a different population [2–4]. These negative epistatic interactions can cause hybrid sterility and reproductive isolation [1, 5, 6].

Genetic incompatibilities are expected to accumulate the longer two taxa diverge independently from each other. This process, which typically extends over hundreds of thousands to millions of years, can be simulated by producing hybrid offspring from species pairs that have evolved in isolation from one another for varying amounts of time. The positive relationship between incompatibility and divergence time has been confirmed experimentally in many different taxa [7–13] and with theoretical models [14–16]. One aspect of the incompatibility-divergence time relationship is that incompatibilities are predicted to increase faster than linearly with divergence time, the so-called ‘snowball effect’ [15]. Two recent studies confirmed experimentally that the number of genes causing inviability increases quadratically (or faster) with their relative divergence times [17, 18].

The main breakdown of hybrid fitness should only be observed after the F1 generation, when recessive D-M factors are increasingly expressed [19, 20]. However, many studies on the relationship between post-mating isolation and time since divergence have used F1 generation hybrids only (e.g. [7, 8, 10, 12, 17, 18]). Assessment of this relationship in higher generation hybrids, especially in animals, is less frequent (but see [11, 21, 22–25]). The intertidal copepod *Tigriopus californicus* is well-studied in this respect. Hybrids of divergent populations show increased fitness with increasing genetic/geographic distance in F1 hybrids, whereas in F2 hybrids the opposite is observed. Fitness decreased significantly with increasing genetic/geographic distance consistent with the D-M model [21]. Then again, a more recent study on the same system demonstrated that consequences of interpopulation hybridization were surprisingly benign for higher generation hybrids [24]. In other cases, negative fitness effects in F2 hybrids were entirely absent, e.g. in farmed x wild salmon hybrids [22], or in hybrids between wild *Arabidopsis* populations [26]. These mixed results demonstrate the importance to investigate beyond the F1 when assessing the long-term fitness consequences of hybridization. An alternative approach to studying hybrid break down in the F2 is to investigate recessive alleles involved in their inviability or sterility (e.g. 6, [27–29]).

Although cichlid fish are one of the best-studied model systems for speciation, data from F2 hybrids on the incompatibility-divergence time relationship are, as yet, missing. Here, we explore the fitness of second generation hybrids of African haplochromine cichlids bred from species pairs with increasing genetic differentiation, spanning several thousand to 2.7/3.8/7.4 million years (MY) divergence time (estimates based on the lower bound of an internally calibrated, linear clock using the age of Lake Malawi [30], and two relaxed non-linear molecular clocks using the cichlid fossil record and the break up of Gondwanaland [31])

This study continues a previous experiment on F1 hybrids [12], which we interbred to produce F2 hybrids, allowing us to compare the inviability rates found in the F1 and F2 generation. We measured hybrid viability at four different life stages from the zygote to early sexual maturity. We expected 1) lower fitness of F2 hybrids compared to both the F1 hybrids and non-hybrid crosses (within parental lineages), and 2) increasing F2 hybrid inviability with divergence time between grandparental lineages. We found both predictions confirmed, which is in agreement with hybrid breakdown in the F2 generation.

## Methods

### Hybrid crosses

Crosses involved seven different cichlid fish species in different pairwise combinations, bred from laboratory populations. F2 hybrids were produced from a total of 14 F1 hybrid families representing seven different cross types (Table 1). The F1 families were the viable and fertile hybrid offspring generated in an earlier study on first generation hybrid fitness [12]. F1 hybrids of six different crosses reached adulthood and reproduced, representing five different genetic distances (Table 1). Only for one cross (*M*. *estherae x A*. *calliptera*), both sexually reciprocal F1 hybrid crosses were available (i.e. *M*. *estherae* males crossed with *A*. *calliptera* females, and *M*. *estherae* females crossed with *A*. *calliptera* males) to produce F2 offspring. For all other crosses, sexually reciprocal matings could not be obtained (probably due to courtship-related behaviours). Fish were maintained at the Eawag Center for Ecology, Evolution and Biogeochemistry Kastanienbaum, Switzerland. For breeding, fish were kept in 100x40x40 cm. All aquaria were part of a large water recirculation system, in which water chemistry, temperature, light conditions, and feeding regime were kept constant over the entire duration of the experiment, including the rearing of grandparental, F1 and F2 crosses. Dry food was provided daily and twice a week fish were fed with a blend of shrimps, peas and *Spirulina* powder. The light regime in the aquaria was 12L:12D and water temperature was kept at 24–26 °C. Stones and plastic pipes were provided for males to become territorial and to motivate courtship. Offspring from non-hybrid (grandparental) crosses were generated and raised contemporaneously with F1 hybrids, but because F2 hybrids were generated from F1 hybrids, F2 data were taken at a later time point. However, any variation in fitness between these groups should be due to differences in their pedigree because rearing conditions were kept identical.

**Table 1:** Experimental crosses with genetic distance, divergence time and sample sizes.

female parent	male parent	geo- graphy	genetic distance	divergence internal clock	divergence fossil record	divergence Gondwana break up	n F1 families fert	n F1 families hatch	n F1 families 14 surv	n F1 families 180 surv	n F1 families cumul	n F1 families to prod. F2	n F2 families fert	n F2 families hatch	n F2 families 14 surv	n F2 families 180 surv	n F2 families cumul
*Pundamilia pundamilia*	*Pundamilia nyererei*	sym	0.007	0.35–0.61	0.104	0.135	-	-	-	-	-	2	3	3	3	4	-
*Pundamilia nyererei*	*Paralabidochr*. *chilotes*	sym	0.007	0.35–0.61	0.104	0.135	2	2	2	2	2	2	9	9	9	7	6
*Metriaclima estherae*	*Astatotilapia calliptera*	sym	0.019	0.93–1.64	0.58	0.919	5	5	5	4	4	2	6	6	5	6	6
*Astatotilapia calliptera*	*Metriaclima estherae*	sym	0.019	0.93–1.65	0.58	0.919	3	3	3	3	3	2	7	7	7	6	3
*Astatotilapia calliptera*	*Protomelas taeniolatus*	sym	0.024	1.19–2.1	0.891	1.485	4	2	2	2	4	2	10	10	10	12	5
*Astatotilapia calliptera*	*Astatotilapia burtoni*	allo	0.041	2.02–3.56	2.226	4.117	14	12	11	10	14	3	10	10	10	12	9
*Astatotilapia calliptera*	*Pundamilia nyererei*	allo	0.055	2.74–4.82	3.779	7.426	14	13	10	10	14	1 (mixed)	10	10	10	10	6
*Pundamilia nyererei*	*Pundamilia nyererei*	sym	0	0	0	0	3	3	3	3	3						
*Pundamilia pundamilia*	*Pundamilia pundamilia*	sym	0	0	0	0	3	3	3	3	3						
*Paralabidochr*. *chilotes*	*Paralabidochr*. *chilotes*	sym	0	0	0	0	3	3	3	3	3						
*Metriaclima estherae*	*Metriaclima estherae*	sym	0	0	0	0	3	3	3	3	3						
*Astatotilapia calliptera*	*Astatotilapia calliptera*	sym	0	0	0	0	5	5	5	5	5						
*Protomelas taeniolatus*	*Protomelas taeniolatus*	sym	0	0	0	0	3	3	3	3	3						
*Astatotilapia burtoni*	*Astatotilapia burtoni*	sym	0	0	0	0	3	3	3	3	3						

Crosses with parental species names, geography (sym = sympatric, allo = allopatric), genetic distance (uncorrected p), and divergence times in million years based on the lower and upper bounds of an internally calibrated, linear clock (using the age of Lake Malawi [30]), and two relaxed non-linear molecular clocks using the cichlid fossil record and the break up of Gondwanaland [31]. For details on these calibrations see Stelkens et al. [12]. The number of families used for the calculation of the different fitness components are shown (fert = fertilization rate, hatch = hatching rate, 14 surv = survival rate until 14 days after hatching, 180 surv = survival rate until 180 days after hatching, cumul = cumulative fitness), and the number of F1 families used to produce F2 (“mixed” indicates one case where we were forced to merge several F1 families before generating F2). Homospecific, non-hybrid crosses are listed in the lower part of the table. Hyphens indicate missing data. The number of families available usually drops with developmental stage because mortality increases with each successive measurement. For some families fitness was measured at later stages but not at earlier stages or vice versa, which is why the number of families is not stable or does not always decrease as the experiment progressed.

All animal work conducted here adhered to the relevant national and international guidelines. The Veterinary Office of the Canton of Lucerne (Switzerland) granted permission to conduct this research (permit number LU04/07). The amount and time of handling of the fish was kept to a minimum to ameliorate suffering and distress. No animal was sacrificed for this study.

### Measuring post-mating isolation

Four different components of post-mating isolation were measured. All were previously measured in the F1 hybrid generation of the same cross types, i.e. involving the same two parental species [12]: fertilization failure, hatching failure and survival failure after 14 and 180 days post hatching. Five days after a spawning had occurred the mouthbrooding female was taken out of the experimental tank and eggs were carefully removed from her mouth. Fertilized (yellow-orange) and unfertilized eggs (white) were counted and divided by the total number of eggs produced (1—fertilized eggs/total number of eggs). All fertilized eggs were then placed into an egg incubator (for details see [12]) and kept there until day 14. Clutches in the incubator were checked daily and the number of hatched and surviving fry was counted to calculate hatching failure (1—number of hatched fry/number of fertilized eggs) and survival failure after 14 days (1—number of survivors at day 14/number of hatched fry). Dead eggs and larvae were removed daily. On day 15, fry were transferred to small Aquaria (20x40x20 cm). At the age of 30 days, fish were moved to bigger tanks (50x40x30 cm), where they were grown until day 180. On day 180, the survivors of a clutch were counted and 180-day survival failure was calculated (1—number of individuals at day 180/number of survivors at day 30). Additionally, we calculated a cumulative measure of survival from egg to day 180 (1—number of survivors at day 180/number of eggs).

Average inviability of homospecific, F1 hybrid and F2 hybrid crosses was calculated by first averaging across replicated F2 clutches bred from the same F1 family, then averaging across replicated F2 families of the same cross type, and then across the different cross types within homospecifics, F1 hybrids and F2 hybrids (for sample sizes see Table 1). F2 inviability per genetic distance was calculated by averaging across cross types of that genetic distance. To compare the inviability of homospecific crosses with the inviability of F1 and F2 hybrids, ANOVA and Student’s t-tests were used after testing if the data fulfill normality assumptions. The alpha critical p-value was adjusted to 0.0033 after Bonferroni correction for multiple testing. Linear regressions were used to test whether genetic distance predicts inviability in F2 hybrids. All analyses were carried out in JMP 10 [32].

### Calculating genetic distances and divergence times

Genetic distances between species are uncorrected p-distances based on D-loop sequences from NCBI GenBank (http://ncbi.nlm.nih.gov/genbank) and were extracted from Stelkens *et al*. [12], where calculations of genetic distances and divergence times is described in detail (see also Table 1 and S1 Table).

## Results

In total, 42 F1 hybrid families, 52 F2 hybrid families, and 23 non-hybrid families were used to compare fitness (Table 1). The F1 data used here, taken from Stelkens *et al* [12], includes 3644 eggs (of which 2788 fertilized), 2208 hatchlings, 1219 14-day old fish, and 858 180-day old fish. The F2 data generated here includes 1560 eggs (of which 1368 fertilized), 902 hatchlings, 567 14-day old fish, and 467 180-day old fish. The homospecific data contains 930 eggs (of which 909 fertilized), 887 hatchlings, 836 14-day old fish, and 557 180-day old fish.

Genetic distances between species ranged from 0.007 to 0.055 and divergence times from several thousand to 2.7/3.8/7.4 million years, depending on the molecular clock (order: lower estimate of the internally calibrated clock / calibration based on fossil records / calibration based on Gondwana break up). Details on all crosses, genetic distances and corresponding divergence times, as well as the number of available F1 families to produce F2, and the number of F1 and F2 hybrid families for the estimation of post-mating isolation components, can be found in Table 1.

### Do hybrids suffer from reduced fitness?

**Fig 1:**
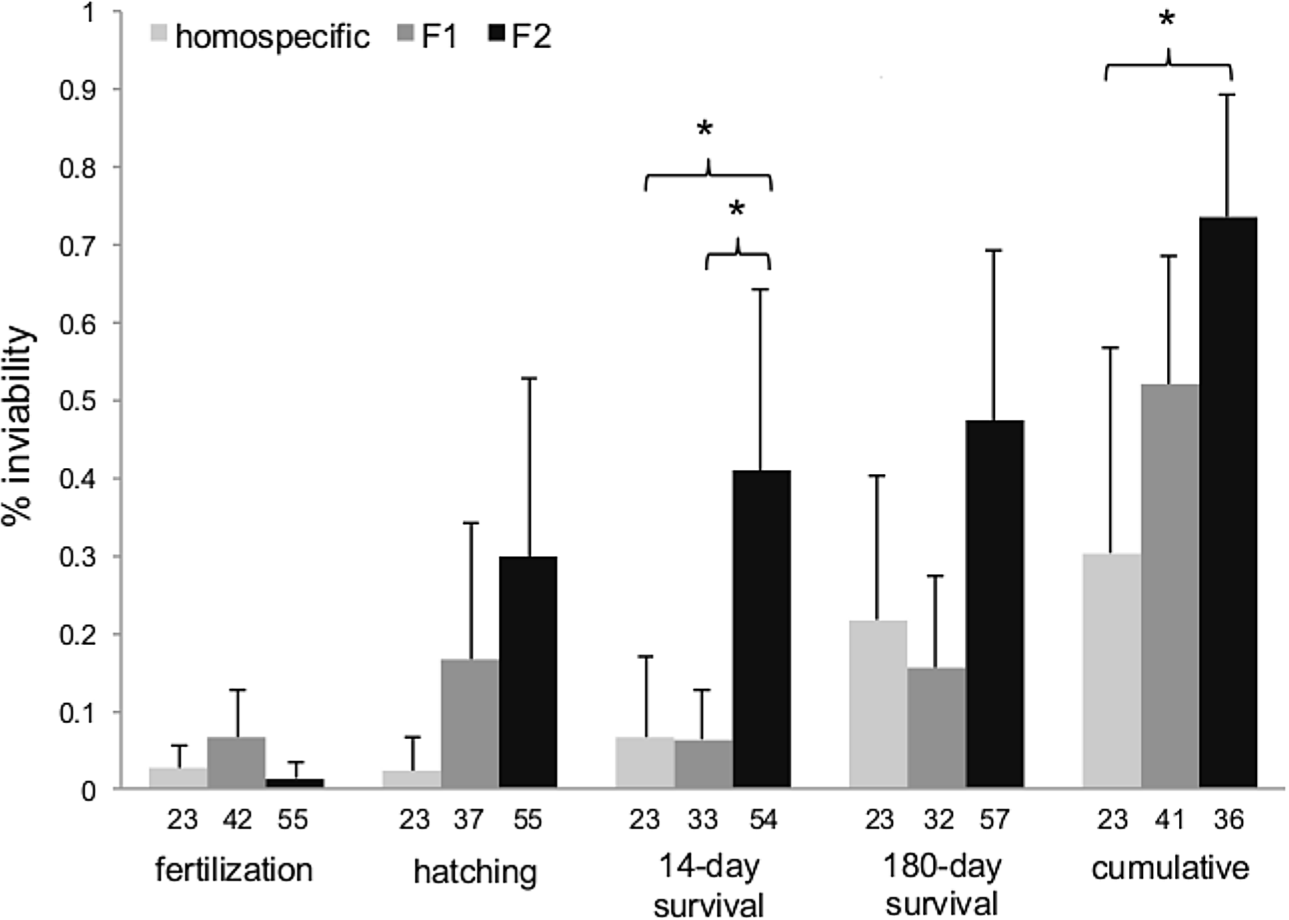
Average inviability of homospecific, F1 and F2 hybrid crosses. Five different measures of post-mating failure rates (in %) in homospecific, F1 and F2 interspecific hybrid crosses. Bars show inviability averaged across replicated F2 families of the same cross type, and then across the different cross types within homospecifics, F1 hybrids and F2 hybrids. Error bars are standard deviations. Numbers under bars represent the number of families entering analysis. Significant pairwise posthoc comparisons (after Bonferroni correction) are indicated by brackets with asterisks.

Homospecific, F1 and F2 hybrid crosses showed significant differences in hatching rate (F_2,20_ = 5.34, p = 0.015), 14-day survival (F_2,20_ = 12.02, p = 0.001), 180-day survival (F_2,20_ = 5.82, p = 0.011) and cumulative fitness (F_2,16_ = 6.73, p = 0.009). Only fertilization rate was not significantly different between groups (F_2,20_ = 3.12, p = 0.069). F2 hybrids consistently showed the highest inviability in all components of fitness, except for fertilization rate (Fig 1).

Pairwise posthoc comparisons showed that, on average, 14-day survival was significantly reduced in F2 hybrids compared to F1 hybrids (after Bonferroni correction; Table 2). Although considerably lower, F2 hatching rates, 180-day survival, and cumulative fitness did not significantly differ from F1 rates. Compared to homospecific (non-hybrid) crosses, F2 hybrids showed significantly reduced 14-day survival, and lower cumulative fitness. F1 hybrids generally showed lower viability, but F1 and homospecific crosses did not significantly differ in any fitness component.

**Table 2:** Results (two-tailed p-values) of Student’s t-tests comparing five components of fitness between homospecific, non-hybrid crosses, F1 hybrid crosses, and F2 hybrid crosses.

Fitness component	homospecific v. F1	homospecific vs. F2	F1 vs. F2
fertilization	0.073	0.561	0.027
hatching	0.12	0.004	0.164
14-day survival	0.982	*0*.*001*	*0*.*001*
180-day survival	0.53	0.014	0.006
cumulative	0.099	*0*.*003*	0.106

Italics indicate significant comparisons after Bonferroni correction.

### Does genetic distance between species reduced fitness in F2 hybrids?

**Fig 2:**
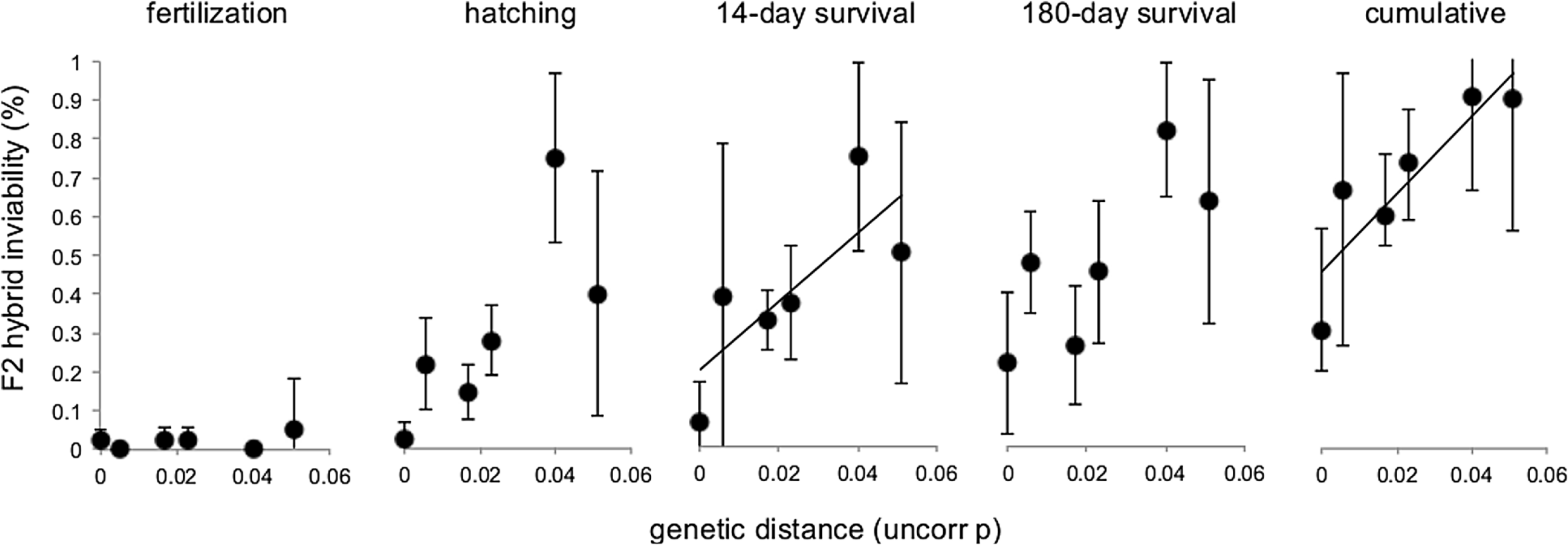
F2 hybrid inviability as a function of genetic distance. Accumulation of fertilization, hatching, 14-day survival, 180-day survival and cumulative failure rates as a function of genetic distance (uncorrected p-distances calculated from D-loop sequences) in F2 hybrids. Error bars are standard deviations. Regression lines indicate significant relationships.

Inviability of F2 hybrids increased with genetic distance between the grandparental populations (Fig 2). 14-day inviability (R^2^ = 0.67, p = 0.046) and cumulative inviability (R^2^ = 0.77, p = 0.022) increased significantly with genetic distance. For hatching (R^2^ = 0.58, p = 0.079) and 180-day inviability (R^2^ = 0.64, p = 0.057) the relationship was strong but non-significant. Fertilization failure was not predicted by genetic distance (R^2^ = 0.21, p = 0.356).

## Discussion

Many studies have investigated the accumulation of genetic incompatibilities in first generation hybrids [7–10, 12, 17, 18, 33, 34]. Generally, these studies report a positive relationship between post-mating incompatibility and genetic distance between the parental species, confirming theory [14, 15]. Assessments of this relationship in higher generation hybrids are less frequent although considerable fitness differences between F1 and F2 hybrids are to be expected because of negative epistatic effects [14]. Although cichlid fish are a model system for many speciation-related questions, data on the fitness of F2 hybrids as a function of grandparental genetic distance is lacking. Here we use African cichlid fish to compare the inviability rates of F2 hybrids with rates found in F1 crosses and the corresponding grandparental-type crosses. We also test whether genetic distance between parental populations predicts an increase in inviability in F2 hybrids.

F2 hybrids consistently showed the lowest viability across all components of fitness (except for fertilization rate). Using a cumulative fitness measure that encompasses all life stages from fertilization to survival to 6 months, F2 hybrids showed a fitness loss of ca. 21% compared to the average fitness of the F1, and a fitness loss of even 43% compared to the grandparental crosses. Especially the short- and long-term survival (2 weeks to 6 months) of post-hatchling F2 hybrids was reduced compared to the survival of F1 and parental crosses. F1 hybrids on the other hand did not suffer from a significant decrease in fitness compared to their parents (though this may not be surprising since we could only use F1 crosses viable enough to produce F2 offspring). Interestingly neither F1 nor F2 hybrids suffered from fertilization failure. In fact, fertilization success in the F2 was significantly higher than fertilization success in F1, indicating that the cell-physiological formation of zygotes does not represent a barrier to gene flow between the species studied here.

Our results are consistent with theory on hybrid breakdown in the F2. Incompatible alleles at different loci, inherited from the parental species, may not be harmful in the heterozygous condition, i.e. in the F1, but can be deleterious when homozygous, a condition, which should be more frequent in higher generation hybrids [3, 19, 20]. In addition, allele segregation can break up beneficial gene combinations in the second generation [35]. With increasing divergence time, and therefore increasing genetic distance, such genetic incompatibilities accumulate as a by-product of the fixation of novel mutations that arose after populations separated, causing postzygotic reproductive isolation. Although not significant for every fitness component that we measured, our data are consistent with this prediction. We found post-hatching mortality rates (from hatching to 14 days) and cumulative F2 inviability (from eggs to 180 days) to increase with the genetic distance between the grandparental populations, suggesting an important role for recessive D-M-incompatibilities in cichlid fish postzygotic isolation. We note that the shape of the relationship we found is not representative of the number of loci involved in the inviability phenotype, but rather of the magnitude of postzygotic isolation [15]. In fact, two recent efforts testing the snowball theory for the rate of evolution of hybrid incompatibilities show that the number of genes causing inviability increases quadratically (or faster) with lineage divergence times in *Drosophila* [17] and *Solanum* [18].

An important limitation of our study with respect to estimating reproductive isolation is that we do not have data on F1 (or F2) hybrid sterility, which could considerably contribute to hybrid breakdown. We acknowledge a further caveat in our study: species divergence times were estimated with a single mitochondrial region (D-loop). Given our knowledge of the ease with which mitochondrial genomes can introgress, it is possible that our estimates of genetic distance underestimate the original splitting between the sympatric species pairs, which would make the increase of incompatibility with divergence less steep. Another source for inaccuracy is the phylogentic dependence of the species pairs used (five out of seven crosses include the same species). Correcting for phylogenetic relatedness did not change the conclusions on the increase of incompatibility with divergence time in F1 hybrids of the same crosses (see Fig 2 in Stelkens *et al*. [12]), but we caution against an overinterpretation of the shape of the relationship here.

Our study was not designed to identify the genetic basis of reproductive isolation, but a specific deformation indicates a possible cause for hybrid failure. The deformation was already observed by Stelkens *et al*. [12] in F1 hybrids and is characterized by a deformed blood vessel connection between the hatchling and the yolk sac. It develops before the free-swimming stage, i.e. within the first 2 weeks post hatching, when most of the losses in our experiment occurred. The deformation looks similar to the *heartstrings* mutation described for zebrafish which has been shown to be caused by a single recessive mutation [36]. *Heartstrings* has also been observed in sunfish hybrids and has been suggested as a candidate for a Dobzhansky–Muller incompatibility locus in vertebrates [37].

### Implications for cichlid fish evolution

There is growing evidence that interspecific hybridization is an important force in evolution, potentially being a source of genetic variation and evolutionary novelty [38, 39]. Molecular phylogenetic studies suggested that hybridization may have played important roles in rapid adaptive radiations, including those of cichlid fish in African lakes [40]. The radiations of Lake Victoria [41], Lake Malawi [42, 43], and Lake paleo-Makgadikgadi [44] were suggested to have been seeded by hybridizing lineages with divergence times of 3 to 7 MY. Yet, experimental tests of the feasibility of hybridization between such lineages had not been carried out beyond the F1. Here, we successfully produced fully viable F2 hybrid offspring from species that began to diverge up to 2.7/3.8/7.4 million years ago. A large fraction of the hybrids reached maturity, but we also found significant mortality, indicating segregation of strong incompatibilities in our experimental hybrid families. This suggests that naturally occurring hybrid populations of these and similar cichlid lineages are likely experiencing strong incompatibility selection [45, 46]. Future work should identify the signatures of such selection in the genomic composition of adaptive radiations. In the natural context, however, hybrids might primarily backcross to parental genotypes rather than breed with other hybrids. Thus, advanced generation hybrid fitness might not be as severely reduced as indicated here, which would mitigate the severity of F2 breakdown, and help explain the extent of hybridization observed in cichlid fish radiations.

## Supporting Information

S1 Table Species used for crosses with Genbank accession numbers. Genbank accession numbers of D-loop sequences used to calculate genetic distances. If no sequences were available for certain species, sequences of closely related species (in brackets) belonging to the same clades characterized by incomplete mitochondrial DNA sorting were used. Data extracted from Stelkens *et al*. [12].
(PDF)
