# Supplementary material for: Hybrid Breakdown in Cichlid Fish
Source: PLoS One. 2015 May 21;10(5):e0127207. doi: 10.1371/journal.pone.0127207 (PMC4440740; doi:10.1371/journal.pone.0127207)
Supplement: S1 Table — Genbank accession numbers of D-loop sequences used to calculate genetic distances. If no sequences were available for certain species, sequences of closely related species (in brackets) belonging to the same clades characterized by incomplete mitochondrial DNA sorting were used. Data extracted from Stelkens et al. [12]. (PDF) [file pone.0127207.s001.pdf]

Supporting Information

S1 Table: Species used for crosses with Genbank accession numbers. Genbank accession numbers of D-loop sequences used to calculate genetic distances. If no sequences were available for certain species, sequences of closely related species (in brackets) belonging to the same clades characterized by incomplete mitochondrial DNA sorting were used. Data extracted from Stelkens *et al*. [12].

Species	Genbank accession numbers
Pundamilia pundamilia / nyererei	AF213546, AF213548, AF213547, AF213529 (*Paralabidochromis plagiodon*)
Paralabidochromis chilotes	AF213540, AF213539, AF213525
Metriclima estherae	AY930025 (*Metriaclima zebra*),<br>AF213620, AY911810, AY911811, AY911812 (*Metriaclima callanois*)
Astatotilapia calliptera	AF298938, AY911722, AY929977, AF298940, AF298939, AF298941, AY911723
Astatotilapia burtoni	AY929999, AF298905, AY929955, AF298906, AY930000, AY930001, AF298904
Protomelas taeniolatus	AF298963, AY913942, EF6475464
